# Substrate-tuning of correlated spin-orbit oxides revealed by optical conductivity calculations

**DOI:** 10.1038/srep27095

**Published:** 2016-06-03

**Authors:** Bongjae Kim, Beom Hyun Kim, Kyoo Kim, B. I. Min

**Affiliations:** 1 Department of Physics, PCTP, Pohang University of Science and Technology, Pohang, 37673, Korea; 2 MPPC CPM, Pohang University of Science and Technology, Pohang, 37673, Korea

## Abstract

We have systematically investigated substrate-strain effects on the electronic structures of two representative Sr-iridates, a correlated-insulator Sr_2_IrO_4_ and a metal SrIrO_3_. Optical conductivities obtained by the *ab initio* electronic structure calculations reveal that the tensile strain shifts the optical peak positions to higher energy side with altered intensities, suggesting the enhancement of the electronic correlation and spin-orbit coupling (SOC) strength in Sr-iridates. The response of the electronic structure upon tensile strain is found to be highly correlated with the direction of magnetic moment, the octahedral connectivity, and the SOC strength, which cooperatively determine the robustness of *J_eff_ *= 1/2 ground states. Optical responses are analyzed also with microscopic model calculation and compared with corresponding experiments. In the case of SrIrO_3_, the evolution of the electronic structure near the Fermi level shows high tunability of hole bands, as suggested by previous experiments.

With recent developments of epitaxial growth technique, substrate-strain engineering has been employed as a very efficient route to control various physical parameters, especially in ABO_3_ transition metal perovskite systems. As the substrate strain modifies the connectivities of the BO_6_ octahedra, such as bond length and bond angle, there occur corresponding macroscopic changes in the symmetry, electronic structure, and magnetic properties^1^,^2^.

Recently, 5*d* oxide systems have emerged as interesting target systems for the substrate engineering. Due to the prominent role of large spin-orbit coupling (SOC) of Ir 5*d* electrons, which is comparable to the strengths of Coulomb correlation (*U*) and bandwidth (*W*), intensive attention has been focused on the Sr-iridates of Ruddlesden-Popper type, Sr_*n*+1_Ir_*n*_O_3*n*+1_. In particular, Sr_2_IrO_4_ (214) with *n* = 1 and SrIrO_3_ (113) with *n* = ∞ are representative systems, which correspond to the insulating and metallic limits, respectively. Both systems have been described based on the *J*_*eff*_ = 1/2 ground states, where the former has a well-separated Mott-gap, while the latter is thought to be a correlated metal^3^,^4^,^5^. The tetragonality of the system, which is a typical tunable parameter in the substrate-strain engineering, is found to be closely correlated to the magnetic-moment direction in 214 systems which have robust *J*_*eff*_ = 1/2 electronic structure^6^. However, for large tetragonal splitting, the deviation from the *J*_*eff*_ = 1/2 ground state has been reported in the recent experiment, casting the question on the range and the condition of the *J*_*eff*_ = 1/2 picture^7^.

Optical experiments for aforementioned Sr-iridates, which act as a direct probe of electronic structures, show two-peak structure near the Mott gap region (see Fig. 1(a,c)). These *α* and *β* peaks were interpreted as arising from the transitions from the occupied *J*_*eff*_ = 1/2 lower Hubbard band (LHB) and *J*_*eff*_ = 3/2 band to the unoccupied *J*_*eff*_ = 1/2 upper Hubbard band (UHB), respectively, as shown in Fig. 1(a). In 214 system, both *α* and *β* peaks are clearly identified, but, in 113 system, only the *β* peak is identified with the additional Drude contribution in the lower energy regime^3^,^8^.

Motivated by the idea of strain engineering for iridate systems, we have investigated epitaxial-strain effects on the electronic structures of two end members of Sr-iridates: Sr_2_IrO_4_ and SrIrO_3_. Our studies are based on the optical conductivity calculated by the *ab initio* band method, which provides the direct comparison with experiments. Also, cluster-based microscopic model calculations are employed to do parameter-wise analysis of optical conductivity. Note that a similar approach was applied to honeycomb iridate systems to successfully explain key experimental findings^9^. Hybrid functional scheme with inclusion of the SOC term is employed, and the results are analyzed and compared with various experimental strain studies on Sr-iridates, especially, with optical experiments^8^,^10^,^11^,^12^,^13^. We have found that the tensile strain on 214 system can effectively tune the strengths of both electronic correlation and the SOC. Strong interplay among the moment direction, the SOC, and the substrate strain in the *J*_*eff*_ = 1/2 ground state is reflected in the optical conductivities as peak shifts or intensity changes of *α* and *β* optical peaks. On the other hand, in semimetallic 113 system, upon strain, the *J*_*eff*_ = 1/2 electronic structure is found to be rather fragile, but low energy physics coming from narrow hole bands is found to be easily tunable.

## Results

### Sr_2_IrO_4_

**Figure 1:**
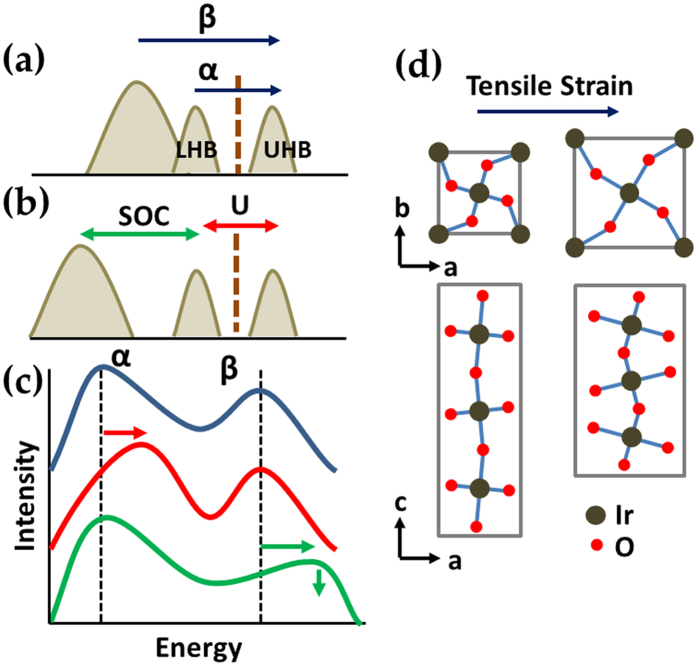
Schematic electronic structures and octahedral connectivity of Sr-iridates. (**a**) Schematic band diagrams of Sr_2_IrO_4_. Optical peaks (*α* and *β*) appear due to the transitions represented by arrows. (**b**) *U* determines the gap between LHB and UHB, while SOC separates *J*_*eff *_= 1/2 and *J*_*eff*_ = 3/2 state. (**c**) The roles of *U* and SOC are schematically drawn. Increasing *U* and SOC shift *α* and *β* peaks, respectively, to high energy sides. (**d**) Tensile strain effects on the IrO_6_ octahedral connectivity of the systems. For 214 systems, due to its layered structure, there is no octahedral connection apically.

Tensile strain increases both Ir-O-Ir angle (*θ*) and Ir-O bond length (*d*) of IrO_6_ octahedron, as shown in Fig. 1(d). The increases in *θ* and *d* play mutually competing roles, as the former enhances the bandwidth (*W*), while the latter localizes 5*d* electrons to increase effective Coulomb correlation (*U*). Recent optical experiment on 214 system showed the systematic shift of *α*-peak with enhanced broadening upon tensile strain^11^. This feature was explained by the enhancements of both *U* and *W*, which increase the separation of UHB and LHB and makes both bands more dispersive, respectively. As typical temperature-dependent behavior shows the enhancement of one parameter with simultaneous suppression of the other, the enhancements of both *U* and *W* are quite unusual^14^.

**Figure 2:**
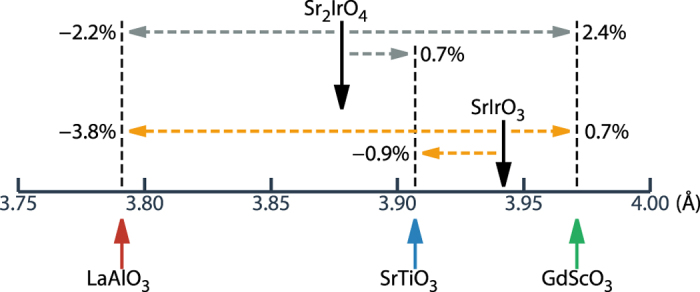
Comparison of in-plane lattice parameter of Sr_2_IrO_4_ (214) and SrIrO_3_ (113) with various oxide substrates. LaAlO_3_ (LAO) and GdScO_3_ (GSO) substrates yield compressive and tensile strains, respectively, while the lattice mismatch would be minor for SrTiO_3_ (STO). In-plane lattices parameters for 214 and 113 systems are from refs 46,47, respectively, where orthorhombic 113 system is converted to corresponding pseudo-cubic phase.

To cover the epitaxial strain range of experimental reports, we have chosen LaAlO_3_ (LAO), SrTiO_3_ (STO), and GdScO_3_ (GSO) substrates. As shown in Fig. 2, LAO and GSO substrates yield compressive and tensile strains, respectively, with +1.9% and −3.2% enhancements of *c*/*a* ratio compared to bulk^11^. In the case of the STO substrate, the lattice mismatch is small, and so the corresponding *c*/*a* ratio change is as small as −0.6%. Optimized *c*/*a* ratio changes of LAO (+1.2%), STO (−2.1%), and GSO (−5.3%) cover well the experimental results. Ir-O-Ir bond angle (*θ*) and Ir-O bond length (*d*) of corresponding 214 systems are summarized in Table 1.

Our calculation results for 214 films demonstrate more prominent role of *U* than *W* upon strain. As shown in Table 2, both spin and orbital magnetic moments systematically increase, as the substrate is changed from LAO to GSO, along with corresponding shifts of optical peaks. In accordance with our results, recent resonant inelastic X-ray scattering (RIXS) experiment observed that the most significant effect of substrate change is the variation of bond lengths, which is manifested in the strengthening (weakening) of the magnetic interaction of the 214 film upon compressive (tensile) strain^12^.

**Figure 3:**
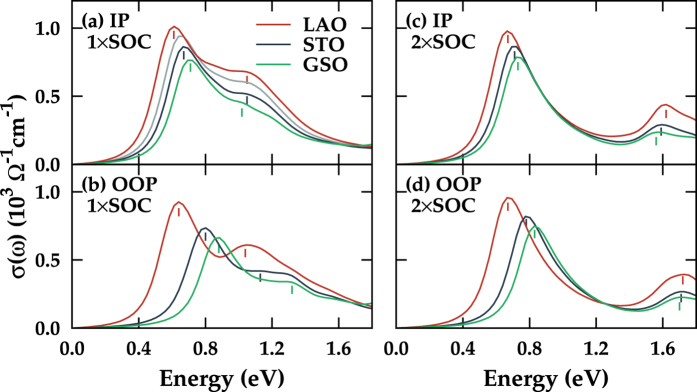
Calculated optical conductivities *σ*(*ω*)'s for Sr_2_IrO_4_ on different substrates. (**a**) IP-AFM ordering cases. Bulk *σ*(*ω*) (gray line) is also given, for comparison. (**b**) Hypothetical OOP-AFM ordering cases. (**c**,**d**) Cases for doubled SOC strength (2 × SOC). At the peak positions, small vertical lines are drawn for the guide to the eyes.

To get the further insight of the role of the strain and to directly compare with the experiments, we have calculated optical conductivity, *σ*(*ω*), using the *ab initio* band methods as described above. Figure 3(a) presents the calculated *σ*(*ω*)’s for 214 system on different substrates. *σ*(*ω*) for bulk is also presented for comparison. Two-peak structure (*α* and *β*) is clearly manifested. Note that, upon tensile strain, the position of *α* peak is shifted to a higher energy side. As schematically depicted in Fig. 1(b,c), this feature is suggestive of the enhancement of effective *U*, which also agrees with the increase in the magnetic moment upon strain (Table 2). In contrast, the *β* peaks are not affected much by the strain, which suggests the different nature between *α* and *β* peaks (see Fig. 1(b,c)). The peak positions of (*α* and *β*) are (0.61, 1.05), (0.67, 1.05), and (0.71, 1.02) eV for LAO, STO, and GSO, respectively, which agree well with existing experiment^11^.

It is seen in Fig. 3 that optical spectrum becomes broadened upon strain. This strain-dependent broadening is interpreted as the increased itinerancy due to change in the bond angle^11^. Despite the prominent role of *U*, as revealed by a shift of the *α*-peak, the broadening of optical spectrum would not be well described in our approach due to lack of dynamical effect^15^. Thus, a possible explanation of broadening in Fig. 3 is that the tensile strain enhances the effective *U*, which reduces the coherency of the electrons. Then, without much change in band width *W*, there occurs broadening of the peaks. The difference between the temperature and the strain dependence of the optical conductivities can be attributed to the altered coherency due to effective *U* variation^14^, which is a subject of further studies.

Due to the two-dimensional (2D) nature of the 214 system, the overall optical responses are composed of in-plane characters only (*σ*_*xx*_ and *σ*_*yy*_). The strain-dependent density of state (DOS), band structure, and hopping parameter are provided in the the supplement materials.

According to the previous studies on *J*_*eff*_ = 1/2 systems, the SOC and the tetragonality are crucial parameters to stabilize the in-plane ordering of the system^6^,^16^,^17^. To investigate the roles of the SOC and the magnetic moment direction in determining the strain-dependent electronic structure of the system, we analyzed *σ*(*ω*)’s (i) for different magnetic moment directions: real in-plane (IP) and hypothetical out-of-plane (OOP) antiferromagnetic (AFM) orderings, and (ii) for normal and enhanced SOC strengths. As discussed below, the OOP configuration is related to the magnetic structure of Sr_3_Ir_2_O_7_ (327) system. In Fig. 3(b), calculated *σ*(*ω*)’s for the OOP case are plotted. Compared to the IP case, the OOP case shows quite different response of the electronic structure to the substrate strain. The overall shifts are very large for the OOP case. As the substrate changes from LAO to GSO, *α* peak positions change by 0.10 eV and 0.24 eV for the IP and the OOP, respectively, while *β* peak positions change by −0.03 eV and 0.28 eV for the IP and the OOP, respectively. Namely, when the 214 system has the IP-AFM ordering, the electronic structure is rather robust against the epitaxial strain, whereas, when the system has the OOP-AFM ordering, the overall electronic structure becomes more susceptible to the strain. In fact, Boseggia *et al*.^18^ linked the IP magnetic ordering in 214 to the *J*_*eff*_ = 1/2 electronic structure, on the basis of its insensitiveness to the structural distortion, which is in agreement with our calculations.

When the SOC strength of the system is doubled (2 × SOC), the most pronounced effect is the large shift-down in energy of *J*_*eff*_ = 3/2 state, as schematically plotted in Fig. 1(b,c), which is reflected by the huge shift-up of the *β* peak in Fig. 3(c). Another notable change is the reduction in the relative intensity of *α* and *β* peak (*I*_*β*_/*I*_*α*_). As each substrate case has different *ω*_*β*_/*ω*_*α*_ value (1.72, 1.57, and 1.44 for LAO, STO and GSO (for 1 × SOC IP case)) and as there is 1/*ω* dependence in the optical conductivity, the intensity is not to be defined by the height of each peak. We have quantitatively analyzed the intensities within a two-peak picture, taking into account the 1/*ω* dependence of the optical conductivity curve, and fitted the data with following Lorentzian-type equation:


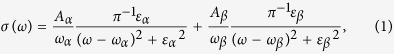


where we can define peak intensity at each frequency position as *I*_*α*_ = *A*_*α*_*π*^−1^/*ε*_*α*_ or *I*_*β*_ = *A*_*β*_*π*^−1^/*ε*_*β*_.

As Kim *et al*.^19^ have shown, the *β* peak, that is thought to arise from transition from low-lying *J*_*eff*_ = 3/2 band to *J*_*eff*_ = 1/2 UHB in a simple picture, has in fact large *J*_*eff*_ = 1/2 LHB contributions. With increasing the SOC parameter, *J*_*eff*_ = 1/2 and *J*_*eff*_ = 3/2 bands are decoupled, and *I*_*β*_/*I*_*α*_ is diminished because of the reduction of *J*_*eff*_ = 1/2 contribution to *β* peak. Namely, the effective increase of the SOC strength can be identified as the decrease of *I*_*β*_/*I*_*α*_. We can clearly see the reduction of *I*_*β*_ with respect to *I*_*α*_ for 2 × SOC cases in Fig. 3(c,d), regardless of moment directions and substrate types (see Table 2).

Surprisingly, *I*_*β*_/*I*_*α*_ ratio is found to decrease systematically upon strain, as shown in Table 2 for different substrate strain cases. This feature suggests that the tensile strain acts similarly to the increased SOC strength. The ratio of orbital and spin magnetic moment (*μ*_*O*_/*μ*_*S*_) also shows similar trend. As the tensile strain is applied, the *μ*_*O*_/*μ*_*S*_ value increases and approaches to 2 (see Table 2), which corresponds to a value for the ideal *J*_*eff*_ = 1/2 state of strong SOC limit. The *β* peak shift, which occurs for increased SOC strength (2 × SOC), has been observed in the experiment^11^, even though it is not identified within our studied substrate-strain range. This feature indicates that the SOC can be enhanced effectively by means of the tensile strain. However, according to the atomic microscopic model, the strain-dependent hopping parameter is also found to produce similar optical behavior for a fixed SOC strength. Thus the overall optical behaviors are expected to come from combined effects of both the SOC strength and hopping parameters. For the OOP-AFM case, upon tensile strain, similar reduction of *I*_*β*_/*I*_*α*_ is obtained, but *μ*_*O*_/*μ*_*S*_ decreases as opposed to the IP case (see Table 2). This feature occurs due to the eventual breakdown of *J*_*eff*_ = 1/2 electronic state rather than the increase in the SOC strength.

Table 3 provides the band gap dependence on the magnetic moment direction in 214 system. Considering that the ideal *J*_*eff*_ = 1/2 picture is validated in the insulating limit, the overall increasing behavior of the band gap upon strain is quite reasonable^20^.

**Figure 4:**
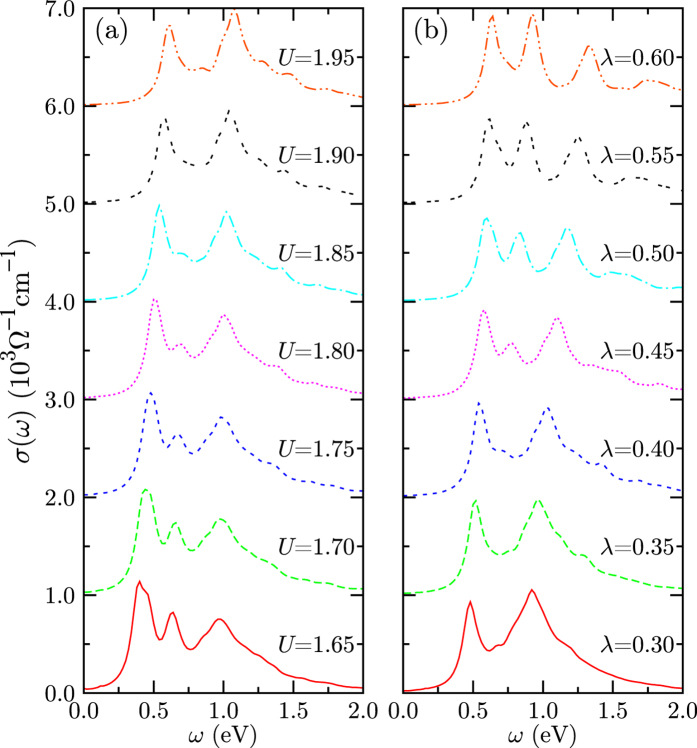
Optical conductivities obtained by four-site cluster multiplet calculations for various on-site Coulomb strengths (*U*) and spin-orbit strengths (*λ*). (**a**) Varying *U* for fixed *λ* = 0.4 eV. (**b**) Varying *λ* for fixed *U* = 1.86 eV. Other parameters are the same as in ref. 19.

To confirm the enhanced *U* and SOC behaviors upon strain, we obtained *σ*(*ω*) using the microscopic model calculations with varying physical parameters. Figure 4(a,b) presents *σ*(*ω*)’s with respect to *U* and *λ*, respectively. Dominant optical spectra are attributed to the electron-hole (e–h) excitations in the vicinity of the Mott gap. With increasing *U*, the optical peaks shift up due to the enhancement of Mott gap. In addition, the shape of optical spectrum varies depending on *U* values. The change from three-peak to two-peak structure is observed. Interesting finding is that the middle-peak is depleted when the shape of optical spectrum changes. It is expected to occur due to the Fano-type coupling between the spin-orbit (SO) exciton and e–h excitation of *J*_*eff*_ = 1/2 band^19^. Whether three-peak structure really appears in *σ*(*ω*) of iridate is not so certain, because the four-site cluster we have considered in Fig. 4 may not be sufficient to describe full kinetics of lattice. However, it is legitimate to infer that some optical spectral-weight transfer to higher peak (*β* peak) occurs with increasing *U*, which corresponds to tensile strain behavior.

When the SOC increases, the splitting between *J*_*eff*_ = 1/2 and 3/2 bands increases and the Mott gap is slightly enhanced. These features are well reflected in the optical conductivity shown in Fig. 4(b). The lowest energy peak becomes slightly higher and the highest energy peak shifts up somehow, when *λ* increases. As in the case of the weak *U* (<1.8 eV), a three-peak structure appears for large *λ* (>0.45 eV). It happens because the Fano-type coupling is weakened as the excitation energy of the SO exciton becomes higher than that of the e–h excitations. Because some spectral weights depleted for small *λ* are recovered for large *λ*, the spectral weight near the *β* peak diminishes when *λ* becomes larger. *I*_*β*_/*I*_*α*_ behavior shows the reduction upon increasing *λ*, which also mimics the tensile-strain effect in the *ab initio*-based optical data, suggesting the effective increase of SOC strength.

Note that, in the current model approach, *I*_*β*_/*I*_*α*_ is also affected by the change in the hopping parameters due to substrate strain, namely, the enhanced hopping between *J*_*eff*_ = 1/2 bands and the reduced hopping between *J*_*eff*_ = 1/2 and *J*_*eff*_ = 3/2 bands, under the tensile strain. Thus, regarding the peak intensities, the enhanced optical spectral weight of e–h excitation of *J*_*eff*_ = 1/2 is expected to yield similar effect to the enhanced SOC strength.

In general, care should be taken for applying low-energy atomic model to itinerant 5*d* system. Since the intensity of *σ*(*ω*) in the model approach is obtained by the sum of possible four spectral weights from *d*^4^–*d*^6^ multiplet configurations^19^, the analysis of the each spectral weight upon parameter change is possible. In the *ab initio* methods however, the strain-dependent change in *I*_*β*_/*I*_*α*_ can be the result of cooperative changes in many physical parameters, not solely from SOC strength. As we have seen in the IP and OOP cases, the additional information on *μ*_*O*_/*μ*_*S*_ change is necessary to conclude that the primary tuning parameter in the IP case is the SOC strength, while it is not in the OOP case.

The opposite behavior of *μ*_*O*_/*μ*_*S*_ for IP and OOP can also be understood in terms of a simple atomic picture. For a state close to ideal *J*_*eff*_ = 1/2 state, *μ*_*O*_/*μ*_*S*_ can be expressed as


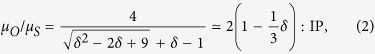



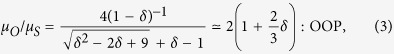


where *δ* = 2Δ/*λ* (*λ*: SOC strength) represents small deviation from the ideal cubic case due to tetragonal crystal field splitting (Δ) (see the supplement materials for the derivation).

Considering itinerant character of 5*d* system, atomic model may not access the full description of the system, but the strain dependency is expected to be well-described. As *δ* goes more negative upon tensile strain, the IP (OOP) case shows clear increase (decrease) in *μ*_*O*_/*μ*_*S*_. The more rapid decrease for the OOP case agrees well with the tendency shown in Eqs (2) and (3) (see Table 2).

The different strain dependence between 214 and Sr_3_Ir_2_O_7_ (327) system are also expected to come from the different magnetic moment directions, as the former and the latter have IP and OOP-AFM orderings, respectively. The strain dependence of 327 system resembles the hypothetical OOP-AFM phase of 214 system^15^, which suggests that the response of electronic structure upon strain is more related to the moment direction than to the dimensionality of the Sr-iridates. The tensile strain can effectively change the *J*_*eff*_ = 1/2 nature of the system through the change of the moment direction as well as the change in the electronic correlation^17^. In conjunction with recent analysis on resonant X-ray scattering of iridate systems, we corroborate that the moment direction plays a role of another degree of freedom that can be tuned using the substrate engineering, especially, for a system with many competing energy scales^21^. Note that the different responses upon strain between the IP and OOP cases can also be viewed as increased anisotropy in the electronic structure. Isotropic *J*_*eff*_ = 1/2 ground state becomes anisotropic due to the crystal field *δ* coming from the strain, and the relatively higher change in the electronic structure shown in the OOP case can be interpreted as stronger dependence on tetragonal distortion *δ* of the electronic structure, which has been shown for *μ*_*O*_/*μ*_*S*_ behaviors (See Eqs (2) and (3)).

The 2 × SOC cases show overall similar strain trends, but with more robustness of the electronic structures. As can be seen by increased *μ*_*O*_/*μ*_*S*_ along with reduced *I*_*β*_/*I*_*α*_ (Table 2), the electronic structure for the 2 × SOC becomes closer to that of *J*_*eff*_ = 1/2 state, which is reflected by highly reduced optical peak shifts upon external strain in Fig. 3(c).

According to the model by Jackeli *et al*.^6^, the direction of magnetic moment of the *J*_*eff*_ = 1/2 system can be switched from IP to OOP by changing the local crystal-field splitting. Indeed, a recent first-principles study^17^ showed that the change of the magnetic order from in-plane to out-of-plane occurs when the ratio of apical and planar Ir-O bond lengths should exceed 1.09. This value, however, is at or beyond the limit of coherent growth of the perovskite oxides through epitaxy. Our substrate strain covers the bond length ratio from 1.045 (LAO) to 0.998 (GSO), and so the stable IP magnetic order is retained for all studied substrate-strain range. This feature is also supported by our energetics study, which provides that the IP-AFM structure is more stable than the OOP-AFM by 100 meV/f.u. In fact, to flop the magnetic moment of 214 system, direct doping of magnetic ion seems to be much more efficient^22^. The substrate tuning approach is expected to be efficient rather for the 327 bilayer system, in which the energy difference between IP-AFM and OOP-AFM is much smaller. The 2 × SOC case shows even larger energy difference between IP-AFM and OOP-AFM, which suggests the strong interconnection between the *J*_*eff*_ = 1/2 electronic structure and the magnetic moment direction of the system.

On the basis of the above studies, it is worthwhile to check the possible magneto-electric effect in 214 system. As the tensile strain increases, the overall electronic structures of 214 system between the IP and OOP cases become progressively distinct, which is revealed by the differences in *I*_*β*_/*I*_*α*_, *μ*_*O*_/*μ*_*S*_, and the optical conductivity shapes for different substrates (Table 2 and Fig. 3). Different electronic structures between IP and OOP moment directions can be utilized to generate strong magneto-electric effect, especially for strained system, as such by applying the strong magnetic field^23^. Namely, the control of the electronic structure, such as optical gap, would be feasible by employing the strained iridate systems.

### SrIrO_3_

Differently from 214 system, 113 system is known as a correlated metal with semimetallic character, being located at the boundary of the magnetic metal and magnetic insulator in the phase diagram^4^,^15^,^24^,^25^. We have found that 113 system is to be a paramagnetic metal for all studied substrate-strain range. In 113 system, the response of the electronic structure to the epitaxial strain is expected to be reduced with respect to the case in 214 system, due to the 3D connectivity of the IrO_6_ octahedra. As shown in Fig. 1(d), in 113 system, the planar strain effects are expected to be compensated by the change in apical connectivity of the IrO_6_ network, which can be seen in the apical and in-plane bond length and bond angle variations upon strain (see Table 4). Accordingly, the overall change of the electronic structure is suppressed, which is in stark contrast to the case in 214 system exhibiting the larger band width variation through direct control of the orthorhombic distortion. Related change of the hopping parameters is presented in the supplement materials.

**Figure 5:**
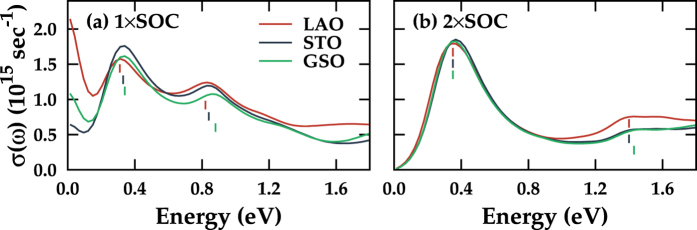
Calculated optical conductivities for SrIrO_3_ (113). Cases with (**a**) normal (1 × SOC) and (**b**) doubled SOC term (2 × SOC) on different substrates. At the peak positions, small vertical lines are drawn for the guide to the eyes.

Optical experiment for 113 system has shown that the *β* peak position is shifted to a higher energy side as the tensile strain is applied, while the *α* peak is not clearly identified^8^. We also obtained the shift of *β* peak by 0.06 eV from LAO to GSO substrate (Fig. 5(a)). The *α* peak, which has not been identified in experiment, appears in our calculation due to the incapability of describing the dynamical correlation effect^15^,^26^. Since the 113 system is weakly correlated, careful change of relative *W* and *U* parameters using substrate strain would produce the correlated three-peak structure in the DOS to locate the *α* peak in the vicinity of the Drude part. Due to 3D connectivity of 113 system, *α* peak shifts are highly suppressed (0.03 eV shift from LAO to GSO case) with respect to the case in 214 system. Note in Fig. 5(a) that, upon tensile strain, the systematic separation of *α* and *β* peaks occurs with the reduction of the *β* peak intensity, as observed in 214 system. According to recent experiments for 113 films, the position of the *β* peak under the small compressive strain shows only a little shift^8^,^13^. Considering that our calculation covers wider range of strain, further experiments with various substrates are demanded to get more information on the substrate effects. Also, recent finding of enhanced scattering for the compressive strain case, which was ascribed to the disorder effect rather than to the correlation effect^27^, can also be justified by examining the *α* peak shift upon substrate strain.

For the 2 × SOC case in Fig. 5(b), much larger shift-up of *β* peaks is shown, as in 214 system. Again, the enhanced *J*_*eff*_ = 1/2 ground state of the system is well-described with highly reduced *I*_*α*_/*I*_*β*_ and with more insulating nature in the DOS. Combined with 3D nature of the system, the enhanced SOC highly stabilizes electronic structure against strain, which is evident from almost locking of both optical peaks in Fig. 5(b). All the substrate cases for 2 × SOC are almost insulating with no Drude contribution in *σ*(*ω*) in agreement with the reported *ab initio* phase diagram^24^.

In the case of 214 system, the IP-AF ordering was essential for the effective tuning of SOC, while the OOP-AF shows the break down of *J*_*eff*_ = 1/2 picture upon strain. For nonmagnetic 113 system, the reduction of *I*_*β*_/*I*_*α*_ cannot be claimed to be due to enhancement of effective SOC (see Table 5). According to recent reports, the ground state of 113 has large deviation from *J*_*eff*_ = 1/2 state and the mixing of *J*_*eff*_ = 1/2 and *J*_*eff*_ = 3/2 is found to be significant with the entrance of octahedral rotations, which is in sharp contrast to layered 214 system^28^,^29^. Since the substrate strain directly changes the octahedral rotations, we can deduce that the shift and reduction of *β* peak in 113 system are due to the deviation of ground state from *J*_*eff*_ = 1/2 state, and *I*_*β*_/*I*_*α*_ reduction is due to enhanced optical spectrum weight of *J*_*eff*_ = 1/2 *e–h* excitation, which is totally different from the case in 214 system.

**Figure 6:**
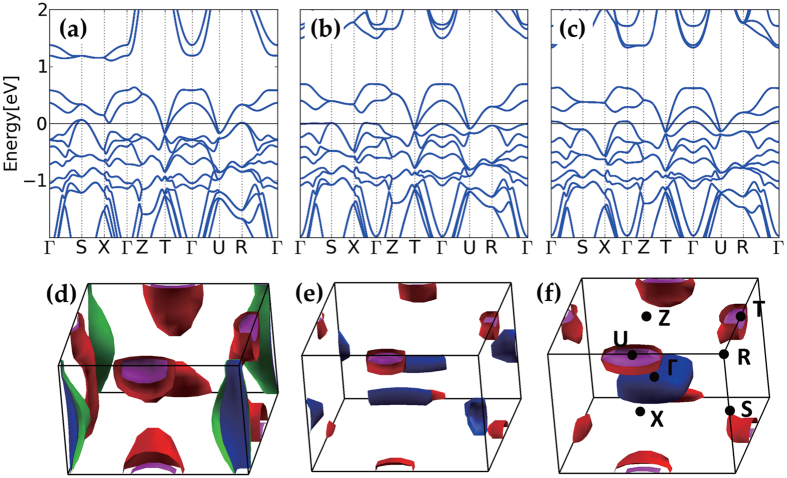
Band structure and Fermi surfaces of 113 system on different substrates (**a**–**c**) band structure for LAO, STO, and GSO substrates. (**d**–**f**) Corresponding Fermi surfaces.

Finally, we want to discuss the low-energy electronic structure of 113 system upon strain. Even though the strain dependency is highly reduced with respect to 214 system due to the dimensionality change, the narrow-band semimetallic nature of 113 system near the Fermi level (*E*_*F*_) makes the system very tunable upon small change of external parameters in low-energy scales. As shown in Fig. 6(a–c), overall band structures of 113 system on different substrates are similar to that of bulk system^24^, but there are a few points to be pointed out. First, we found that 113 system on STO has almost cubic electronic structure, which can be recognized by the highest *e*_*g*_ band location near 2 eV above *E*_*F*_. The tetragonal crystal field in the presence of the substrate strain lifts the degeneracy of *e*_*g*_ states with lowering one out of two *e*_*g*_ states (*z*^2^ for LAO and *x*^2^ − *y*^2^ for GSO) toward *E*_*F*_. Second, while the electron pockets are retained at **k** = *T* and *U*, hole pockets emerge at different ***k***’s depending on the strain, *i.e.* at **k** = *S* and *R* for LAO and near **k** = Γ for GSO. For the STO case, the morphologies of hole pockets are in-between LAO and GSO cases, with very narrow band character near Γ-*S* and *R*-Γ, which enables easy tune upon the epitaxial strain. The Fermi surface topology also changes accordingly, as shown in Fig. 6(d–f).

In relation to recent experiments, heavier effective mass of hole carriers than electron carriers^8^,^28^ can be identified from the band structure of STO substrate case (Fig. 6(b)). More symmetric electron-hole band structure for tensile strain case is also consistent with transport measurement^8^. Under compressive strain, electron pockets at *U* and *T*, and hole pocket at *R* are formed, as shown in Fig. 6(a,d), which are in good agreement with angle-resolved photoemission spectroscopy (ARPES)^29^.

In view of small band renormalization factor of 1.25 from ARPES experiment for 113 system^29^, our results successfully explain the low-energy electronic structure for both compressive and tensile strain cases, and suggest further possibility of manipulating the strain engineering. Inconsistency of simple tight-binding model with ARPES may come from the highly susceptible low-energy electronic structure of the system^29^,^30^. Suggested Dirac-cone-like nodes at *U* and *T* from tight-binding calculation were not detected in the recent ARPES measurement^29^,^31^. Our band structure shows the protected node at *T* upon strain but no Dirac-node at *U*, which needs confirmation by further experimental studies. As the band structure of 113 system depends highly on the *U* value, which is interconnected to the SOC strength, a proper estimation of electronic correlation *U* value is crucial from the theory side^31^,^32^. Also, recent study on 113 film demonstrated that the breaking of the crystal symmetry upon strain can lift the Dirac node of 113 system^33^, which reflects that the electronic structure of the system is highly tunable upon systematic epitaxial strain.

## Conclusion

We have analyzed the substrate strain effects in Sr-iridate systems, employing both the *ab initio* optical conductivity calculation and the microscopic model approach. By analyzing optical peak positions and relative intensities along with obtained magnetic moment, we have found that, in layered 214 system, tensile strain can effectively tune the electronic correlation strength *U* as well as the SOC strength. The robustness of the *J*_*eff*_ = 1/2 electronic structure, which is found to be highly correlated with the magnetic moment direction of the system, can also be controlled by employing the substrate strain effect. On the other hand, in 113 system, tensile strain easily breaks the overall *J*_*eff*_ = 1/2 ground state, and band topology shows highly tunable hole character in the vicinity of *E*_*F*_. Our systematic study demonstrate that the strain engineering for iridate systems, in which various energy scales compete, provides an additional degree of freedom of tunable parameters, *U* and SOC, as shown as peak and weight change of the optical conductivity, which can offer new dimensions on top of the current epitaxial strain studies, especially, when combined with very recent studies based on superlattice structures^34^.

## Methods

### *Ab initio* calculation

We have performed electronic structure calculations, employing the full-potential linearized augmented plane wave (FLAPW) band method^35^,^36^ implemented in WIEN2k package^37^. For the exchange-correlation energy functional, we used the local density approximation (LDA), which has been generally employed for 5*d* systems. To treat the correlation in functional level, we employed the hybrid-functional^38^,^39^, which is given by


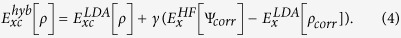


Here Ψ_*corr*_ and *ρ*_*corr*_ correspond to the Kohn-Sham wave function and the electron density of correlated electrons, respectively. The exchange-correlation energy functional is constructed with the fraction (*γ*) of the Hartree-Fock (HF) exchange energy, replacing the LDA correspondence for correlated electrons (5*d*-electrons in the present case). This functional form is the LDA correspondence of so-called PBE0^40^,^41^. Compared to the normally employed LDA+*U* method, the hybrid-functional approach can treat the correlation effects of different systems in a consistent way and the non-local exchange energy can be included in the HF term. The hybrid-functional scheme has been employed for numerous transition-metal (TM) perovskites, from 3*d* to 5*d* systems, and is thought to be one of the best computational schemes^42^. Especially for more itinerant 5*d* systems, recent calculation found hybrid functional scheme successfully described the electronic structures and magnetic properties^43^. The important SOC term is included in the second variational scheme.

To determine the proper *γ* parameter, we performed the calculations on bulk Sr_2_IrO_4_ (214) system with various *γ* values, using both the LDA and PBEsol functionals. As shown in Table 6, both functionals show similar results of increasing gap size with *γ*. Considering the observed optical gap size of around 0.4 eV, *γ* value in-between 0.20 and 0.25 looks appropriate. In the present study, we chose the LDA functional with *γ* = 0.20 to fit the observed optical peak positions. However, the overall strain dependency is expected to be similar for various *γ* values. Our choice of *γ* = 0.20 is somewhat smaller than the often-used typical value of *γ* = 0.25. But the systematic studies for the perovskite systems showed that the smaller value of *γ* produces much better results^42^.

The substrate strain effects were taken into account by fixing in-plane(IP) lattice parameters of 214 and SrIrO_3_ (113) systems to those of the substrates: LaAlO_3_ (LAO), SrTiO_3_ (STO), and GdScO_3_ (GSO). Since the relevant optical experiments were performed not on ultrathin films, we did not consider the substrate materials explicitly. We assumed the collinear magnetic structures for both IP and out-of-plane (OOP) cases based on the fact that the IP ferromagnetic (FM) component due to the canted antiferromagnetic (AFM) structure is substantially weakened for the film case^44^.

We optimized *c*/*a* ratio first with fixed *a*, which determines tetragonality of the system, and then performed the internal relaxations for given volume of every systems with force criteria of 1.0 mRy/a.u. within the LDA limit. With obtained structures, we performed the hybrid-functional calculations with the inclusion of the SOC term. In a system where the SOC plays a dominant role, inclusion of non-diagonal parts of the spin density matrices are found to be crucial. Especially, for iridates, inclusion of only diagonal parts does not describes the energy gap and magnetic moments of the system, which even changes the energetics of the 214 system. Without non-diagonal parts, the magnetic moment direction of the system is found to be OOP, which is corrected only after the inclusion of the full matrix elements. In addition to hybrid functional parts, we included non-diagonal elements of density matrices corresponding *U* = 2 eV in generating orbital potentials, for the description of weakly correlated Ir 5*d* electrons. The valence wave functions inside the muffin-tin spheres were expanded with spherical harmonics up to *l*_*max*_ = 10. The wave function in the interstitial region was expanded with plane waves up to *K*_*max*_ = 7.0/*R*_*MT*_, where *R*_*MT*_ is the smallest muffin-tin sphere radius. *R*_*MT*_ were set as 2.3, 2.1, and 1.5 a.u. for Sr, Ir, and O, respectively. The charge density was expanded with plane waves up to *G*_*max*_ = 12 (a.u.)^−1^. We have used 1000 ***k*** points inside the first Brillouin zone for both 214 and 113 systems.

Optical conductivity is calculated with the WIEN2k optical package with much denser ***k*** points up to 3000 45. The dielectric function is calculated using the following expression:


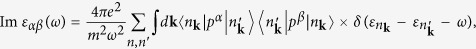


where the transition matrix of the momentum operator *p*^*α*^ between Kohn-Sham states represented by band index *n* and crystal momentum ***k*** with energy 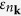 is evaluated and summed. The optical conductivity can be obtained from the Kramer-Kronig transformation,


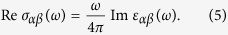


For metallic 113 system, the Drude contribution of the following form is considered,


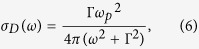


where Γ is lifetime broadening and *ω*_*p*_ is the plasma frequency given by


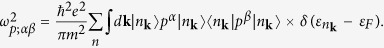


We adopted the Gaussian broadening parameters of the interband transition of the value of 0.10 eV. For the Drude contribution in 113 system, we set 0.10 eV for broadening parameter to describe metallic and semimetallic characters of the systems.

### Microscopic model calculations

Based on four-site cluster calculation including all possible Ir multiplets among *d*^5^−*d*^5^−*d*^5^−*d*^5^ and *d*^4^−*d*^6^−*d*^5^−*d*^5^ charge configurations, we have solved the effective magnetic Hamiltonian with the exact diagonalization (ED) method. *σ*(*ω*) is obtained from the following expression:


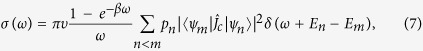


where *υ* is the volume per Ir site, *p*_*n*_ is probability density of eigenstate |*ψ*_*n*_〉, and 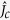 is current operator. See ref. 19 for the details of calculation method and parameters.

## Additional Information

**How to cite this article**: Kim, B. *et al*. Substrate-tuning of correlated spin-orbit oxides revealed by optical conductivity calculations. *Sci. Rep.* **6**, 27095; doi: 10.1038/srep27095 (2016).

## Supplementary Material

Supplementary Information

## Figures and Tables

**Table 1:** Calculated Ir-O-Ir bond angle (*θ*), Ir-O bond length (*d*) of 214 system on different substrates.

	LAO	STO	GSO	bulk
Ir-O-Ir angle (°)	152	156	159	157
Ir-O length (Å)	1.95	2.00	2.02	1.98

Bulk results are also given for comparison.

**Table 2:** Calculated spin, orbital magnetic moments, their ratio, and peak intensity ratio (*μ*_*S*_, *μ*_*O*_, *μ*_*O*_/*μ*_*S*_, and *I*_*β*_/*I*_*α*_) for 214 system on different substrates.

		LAO	STO	GSO	bulk
1 × SOC					
IP	*μ_S_*	0.17	0.18	0.18	0.19
	*μ_O_*	0.24	0.27	0.28	0.26
	*μ_O_*/*μ_S_*	1.43	1.52	1.58	1.40
	*I*_*β*_/*I*_α_	1.31	1.08	1.04	1.22
OOP	*μ_S_*	0.26	0.36	0.40	0.29
	*μ_O_*	0.32	0.38	0.41	0.34
	*μ_O_*/*μ_S_*	1.24	1.06	1.00	1.18
	*I*_*β*_/*I*_α_	1.35	1.09	0.85	–
2 × SOC					
IP	*μ_S_*	0.16	0.16	0.16	
	*μ_O_*	0.25	0.28	0.30	–
	*μ_O_*/*μ_S_*	1.52	1.70	1.81	
	*I*_*β*_/*I*_α_	0.98	0.72	0.68	–
OOP	*μ_S_*	0.21	0.28	0.31	
	*μ_O_*	0.28	0.32	0.32	–
	*μ_O_*/*μ_S_*	1.33	1.14	1.05	
	*I*_*β*_/*I*_α_	0.97	0.66	0.63	–

*I*_*β*_/*I*_*α*_ here is defined by *A*_*β*_*ε*_*α*_/*A*_*α*_*ε*_*β*_, as described in Eq. (1) and below. Bulk results are also given for comparison. Unit of *μ*_*S*_ and *μ*_*O*_ is *μ*_*B*_/Ir.

**Table 3:** Band gaps (in eV) of 214 system on different substrates, depending on the SOC strength and magnetic moment direction.

	moment direction	LAO	STO	GSO	bulk
1 × SOC	IP	0.14	0.28	0.34	0.21
	OOP	0.19	0.44	0.57	0.28
2 × SOC	IP	0.28	0.40	0.46	–
	OOP	0.47	0.49	0.58	–

Bulk results are also given for comparison.

**Table 4:** Calculated Ir-O-Ir bond angle (*θ*), Ir-O bond length (*d*) of 113 system on different substrates.

		LAO	STO	GSO
Ir-O-Ir angle (°)	apical	160	155	152
	in-plane	150	153	155
Ir-O length (Å)	apical	2.06	2.02	1.99
	in-plane	1.96	2.01	2.04

**Table 5:** *I*_*β*_/*I*_*α*_ of 113 systems on different substrates.

	LAO	STO	GSO
1 × SOC	2.59	1.96	1.89
2 × SOC	1.21	0.90	0.92

**Table 6:** Band gap dependence on the size of mixing parameter *γ* for LDA and PBEsol functionals.

functional	*γ *= 0.15	*γ *= 0.20	*γ *= 0.25
LDA (eV)	metal	0.21	0.37
PBEsol (eV)	metal	0.26	0.41

Calculations were done for the experimental bulk Sr_2_IrO_4_ (214) system.
