# Supplementary material for: Substrate-tuning of correlated spin-orbit oxides revealed by optical conductivity calculations
Source: Sci Rep. 2016 Jun 3;6:27095. doi: 10.1038/srep27095 (PMC4891771; doi:10.1038/srep27095)
Supplement: Supplementary Information [file srep27095-s1.pdf]

Supplementary Material:
Substrate-tuning of correlated spin-orbit oxides revealed by optical conductivity calculations

Bongjae Kim[1],* Beom Hyun Kim[1],† Kyoo Kim[1,2], and B. I. Min[1‡]
[1] *Department of Physics, PCTP, Pohang University of Science and Technology, Pohang, 37673, Korea*
[2] *MPPC_CPM, Pohang University of Science and Technology, Pohang, 37673, Korea*

I. COMPONENT-WISE OPTICAL CONDUCTIVITY

Epitaxially strained 214 systems have tetragonal structure with orthorhombic magnetic symmetry. The optical conductivity tensor has only nonzero diagonal components (σ_{xx}, σ_{yy}, and σ_{zz}) with strong two-dimensional character. Thus there is negligible contribution from σ_{zz} component (Fig. S1(a)-(c)).

113 systems have tetragonal structure with nonmagnetic ground state. The overall contributions of the IP and OOP components are systematically changed, as shown in Fig. S1(d)-(f).

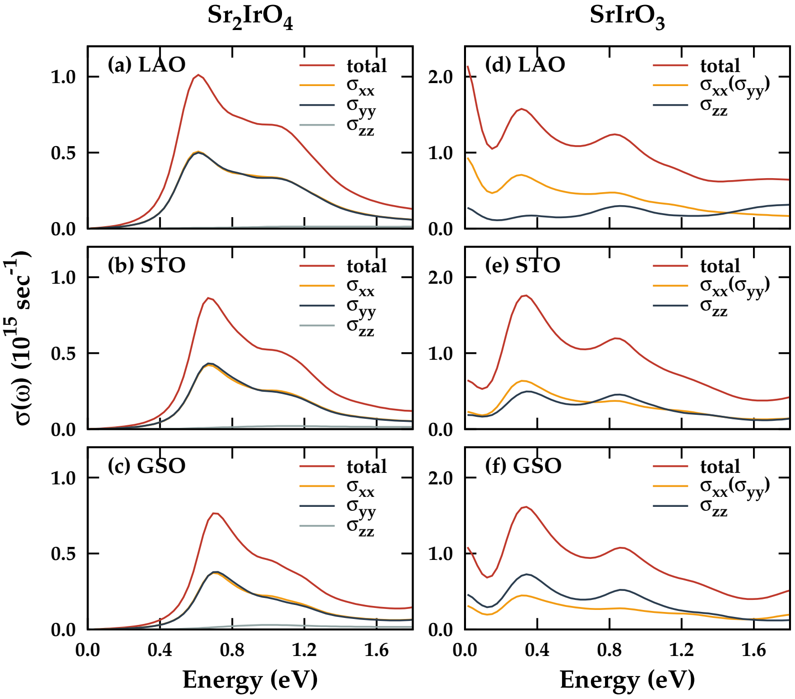

FIG. S1: **Calculated optical conductivities for Sr_2IrO_4 and $SrIrO_3$ with different substrates.** Each case is plotted with diagonal components of conductivity tensor.

II. PARTIAL DENSITY OF STATES

The partial densities of states (DOSs) of Ir-d are shown in Fig. S2. In 214 system, one can clearly see the enhancement of localized character upon tensile strain, as revealed by the sharpening of the DOS. Also, for the 2×SOC case,

*Present address: University of Vienna, Faculty of Physics and Center for Computational Materials Science, Sensengasse 8, A-1090 Vienna, Austria
†Present address: RIKEN, Wako, Saitama 351-0198, Japan
‡bimin@postech.ac.kr

clear shift-down of d-states is shown, as expected from the enhanced separation of J_{eff}=1/2 and J_{eff}=3/2 bands. Overall behaviors in 113 system are similar to those in 214 system.

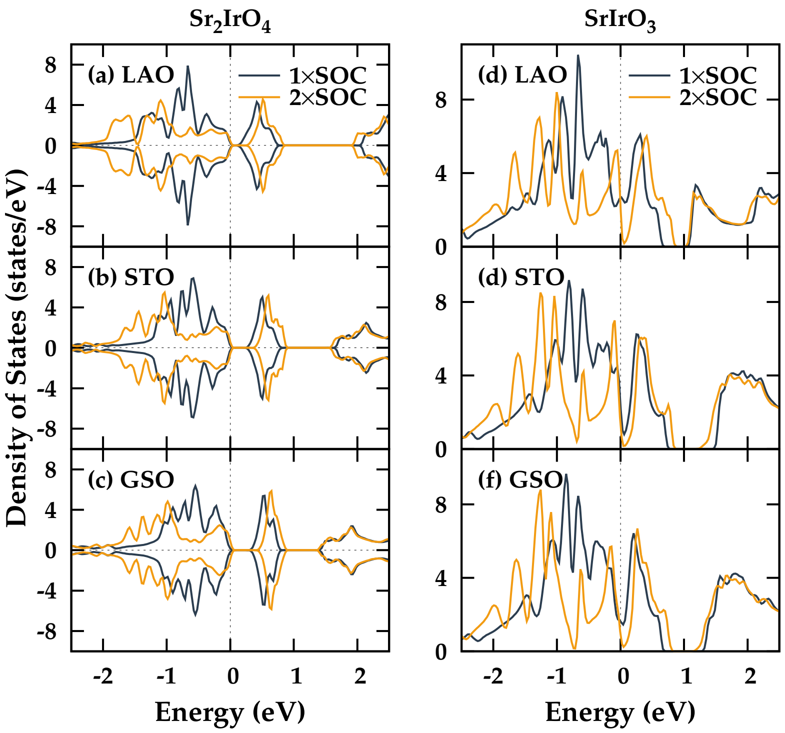

FIG. S2: **Ir-d partial densities of states (DOSs) of Sr_2IrO_4 and $SrIrO_3$ for different substrates.**

III. BAND STRUCTURES AND HOPPING PARAMETERS ON DIFFERENT SUBSTRATES

To examine the dependence of the electron hopping parameters upon substrate strain, we have plotted band structures of the 214 and 113 systems, employing the local-density approximation (LDA) without the inclusion of the electron correlation and the SOC term [S1, S2]. As shown in Fig. S3 for 214 system, one can clearly see the narrowing of the overall band width of the Ir-d t_{2g} bands, which is in accordance with the enhanced localization observed in optical conductivity data. For more quantitative analysis, we have determined the hopping parameters, adopting the Wannier functions approach in the Wannier90 code with an interface to VASP [S3–S5]. As provided in Table S1, the hopping parameters also show gradual reduction as the system is tensile-strained. Due to the absence of the apical connectivity of IrO_6 octahedra, the substrate strain directly affects the electronic structure of the 214 system.

As provided in Table S2 for 113 system, one can observe the decrease (increase) of the hopping parameters along the planar (apical) direction, as the system is tensile-strained. One can also identify narrowing of the band width (Fig. S4), which is also consistent with the optical conductivity calculations. The overall change of the hopping parameters in 113, however, is smaller than that in 214, since the planar and apical changes are compensating each other due to 3D nature of the 113 system.

TABLE S1: Average hopping parameters (t=$\overline{t_{ii}}$, i=d_{xy}, d_{yz}, and d_{zx}) of 214 system on different substrates.

	LAO	STO	GSO
t_{ii} (eV)	−0.19	−0.17	−0.14

TABLE S2: Average hopping parameters (t=$\overline{t_{ii}}$, i=d_{xy}, d_{yz}, and d_{zx}) of 113 system on different substrates.

	LAO	STO	GSO
planar t_{ii} (eV)	−0.19	−0.18	−0.17
apical t_{ii} (eV)	−0.15	−0.17	−0.18

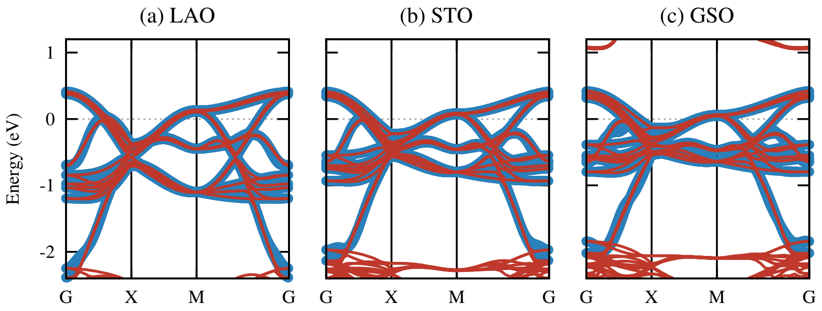

FIG. S3: **Band structures of Sr_2IrO_4 on different substrates.** LDA band structures and Wannier-interpolated t_{2g} bands are shown with red and blue colors, respectively.

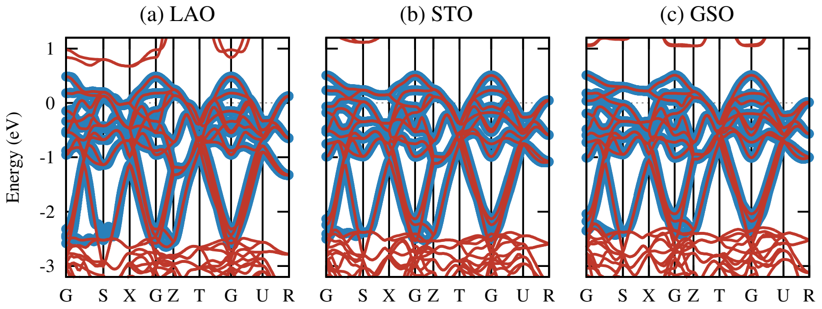

FIG. S4: **Band structures of $SrIrO_3$ on different substrates.** LDA band structures and Wannier-interpolated t_{2g} bands are shown with red and blue colors, respectively.

IV. MAGNETIC MOMENT DEVIATION AT AROUND J_{eff}=1/2 UPON STRAIN

Following the model by Jackeli and Khaliullin,[S6] J_{eff}=1/2 isospin doublet is expressed as:

$$|\tilde{\pm}\rangle = \pm \sin\theta|0,\pm\rangle \mp \cos\theta|\pm 1,\mp\rangle, \quad (1)$$

where θ denotes parameterized angle incorporating tetragonal crystal field splitting ($\Delta = E_{xy} - E_{yz/zx}$) and SOC ($\lambda$) as $\tan(2\theta) = 2\sqrt{2}\lambda/(\lambda - 2\Delta)$. The expected spin and orbital magnetic moments are given by

$$\text{OOP}: \quad \mu_S = \cos^2\theta - \sin^2\theta, \quad (2)$$

$$\text{OOP}: \quad \mu_O = \cos^2\theta, \quad (3)$$

$$\text{IP}: \quad \mu_S = \sin^2\theta, \quad (4)$$

$$\text{IP}: \quad \mu_O = \sqrt{2}\cos\theta\sin\theta, \quad (5)$$

where μ_S and μ_O in OOP (IP) are calculated with $\langle 2s_z\rangle$ ($\langle 2s_x\rangle$) and $-\langle l_z\rangle$ ($-\langle l_x\rangle$), respectively. We can get following μ_O/μ_S ratios

$$\mu_O/\mu_S = \frac{1}{1-\tan^2\theta} = \frac{4(1-\delta)^{-1}}{\sqrt{\delta^2 - 2\delta + 9} + \delta - 1} : \text{OOP} \quad (6)$$

$$\mu_O/\mu_S = \frac{\sqrt{2}}{\tan\theta} = \frac{4}{\sqrt{\delta^2 - 2\delta + 9} + \delta - 1} : \text{IP}, \quad (7)$$

where $\delta = \frac{2\Delta}{\lambda}$ and $\delta < 1$. Cubic case corresponds to $\delta = 0$ and then μ_O/μ_S becomes 2 for any direction. When the strain is applied, there is a deviation (δ). Positive (negative) δ always gives rise to the decrement (increment)

of μ_O/μ_S from 2 for IP. In contrast, μ_O/μ_S for OOP increases (decreases) with positive (negative) δ. Unless other correlation effects modify local electronic structure, δ can be more negative when the tensile strain becomes stronger. μ_O/μ_S ratio is expected to be larger (smaller) in the IP (OOP) case. Note that each tensile and compressive strain corresponds to $\delta < 0$ and $\delta > 0$.

[S1] G. Kresse and J. Hafner, Phys. Rev. B **47,** 558 (1993).
[S2] G. Kresse and J. Furthmüller, Phys. Rev. B **54,** 11169 (1996).
[S3] N. Marzari and D. Vanderbilt, Phys. Rev. B **56,** 12847 (1997).
[S4] A. A. Mostofi, J. R. Yates, Y.-S. Lee, I. Souza, D. Vanderbilt, and N. Marzari, Comput. Phys. Commun. **178,** 685 (2008).
[S5] C. Franchini, R. Kováčik, M. Marsman, S. Sathyanarayana Murthy, J. He, C. Ederer, and G. Kresse, J. Phys.: Condens. Matter **24,** 235602 (2012).
[S6] G. Jackeli and G. Khaliullin, Phys. Rev. Lett. **102**, 017205 (2009).
